# Prevalence of* Haemonchus contortus* Infections in Sheep and Goats in Nyagatare District, Rwanda

**DOI:** 10.1155/2018/3602081

**Published:** 2018-09-06

**Authors:** Borden Mushonga, Dismas Habumugisha, Erick Kandiwa, Oscar Madzingira, Alaster Samkange, Basiamisi Ernest Segwagwe, Ishmael Festus Jaja

**Affiliations:** ^1^School of Veterinary Medicine, Faculty of Agriculture and Natural Resources, University of Namibia, P. Bag 13301, Pioneerspark, Windhoek, Namibia; ^2^School of Animal Sciences and Veterinary Medicine, College of Agriculture, Environment and Veterinary Medicine, University of Rwanda, P.O. Box 57 Nyagatare, Rwanda; ^3^Department of Biomedical Sciences, Faculty of Medicine, University of Botswana, P. Bag UB0074, Gaborone, Botswana; ^4^Department of Livestock and Pasture Science, University of Fort Hare, P. Bag X1314, Alice, South Africa

## Abstract

This study investigated the overall prevalence of* Haemonchus contortus* infection in sheep and goats from five purposively selected subdivisions (sectors) of Nyagatare district from January to December 2014, after a high prevalence of gastrointestinal parasites and generalized poor productivity was reported in small ruminants in some districts of Rwanda. Faecal egg counts (FEC) were performed using the Modified Wisconsin Sugar Floatation method and the Fluorescent-labeled peanut-lectin agglutination test while enumerations, as log (FEC), were done using the modified McMaster method. The overall prevalence of* H. contortus* infection in sheep and goats was 75.7% (n=949). The overall prevalence of* H. contortus* infection in sheep (83.4%, n=314) was higher than in goats (71.8%, n=635) (Odds Ratio [OR] 1.98, 95% Confidence Interval [CI]: 1.40-2.79, and p≤0.001). The prevalence of* H. contortus* infection in female goats (74.2%) was higher than in male goats (64.3%) (OR 1.60, 95% CI: 1.09-2.36, and p=0.01). The prevalence of* H. contortus* infection in goats from Nyagatare was higher than in goats from Matimba (OR 3.25, 95% CI: 1.76-5.99, and p≤0.001) and from Katabagemu (OR 3.67, 95% CI: 2.04-6.59, and p≤0.001). The prevalence of* H. contortus* infection in goats from Karangazi was higher than in goats from Matimba (OR 4.72, 95% CI: 2.40-9.28, and p≤0.001). The overall mean monthly log (FEC) for* H. contortus *in sheep and goats were highest in April (18.9±0.2 and 14.05±0.1, respectively) and October (19.25± 0.2 and 13.75±0.1, respectively). Though, overall, sheep in Nyagatare district were at greater risk of* H. contortus* infection and goats from Nyagatare and Karangazi sectors were paradoxically at greater risk of* H. contortus* infection. It was also apparent that young female goats were at greater risk of* H. contortus *infection than young male goats.* H. contortus* infection is endemic in small ruminants in Nyagatare district and possibly other districts in Rwanda. Targeted selective treatment (TST) using FAMACHA with emphasis on low-lying swampy pastures and appropriate anthelmintic drugs may be the most economically viable solution in the short term. In the long term breeding of* H. contortus* resistant small ruminants and strategic grazing using the concept of refugia may bring about considerable relief from* H. contortus* infection in Nyagatare district, in particular, and Rwanda at large.

## 1. Introduction

Due to their hardiness and fecundity, small ruminants play a crucial role in the cultural and socioeconomic lives of rural people in Africa, providing meat, milk, income, and wealth [1–4].

Helminth infections are one of the most important diseases limiting livestock production in many parts of the world including Rwanda [5–8]. Gastrointestinal helminthes cause significant economic losses of small ruminant enterprises through increased susceptibility of animals to other infections, morbidities, and mortalities, especially in heavily parasitized animals and in young animals. Furthermore, they lead to forced culling, carcass, and organ condemnations [9], increased cost of veterinary treatments, decreased weight gain, reduced milk production and reproductive capacity, decreased work capacity, diminished food intake, reduced animal growth rates, and lower weight gains and treatment and management costs [8–12]. Among these gastrointestinal helminths, nematodes are the leading cause of ill-health and production losses in sheep and goats worldwide [7, 10, 13]. It would appear that a number of reports point to Trichostrongylid nematodes, especially* Haemonchus. contortus *(*H. contortus*) (also known as the barber's pole worm) as the most prevalent parasite identified in small ruminants [8, 10, 11]. According to some reports,* Haemonchus contortus* is the most important gastrointestinal parasite in sheep [5, 14]. However, in a survey in South Eastern Ethiopia* Haemonchus* spp. was not found [15].

*H. contortus* is a helminth parasite of the abomasum of ruminants and, in particular, sheep and goats. It is the most economically important and pathogenic helminth parasite of small ruminants in the warm tropical and subtropical regions of the world [5, 8, 13, 16, 17]. Acutely infected sheep and goats exhibit pale mucous membranes, dark colored faeces, weakness, and edema (bottle jaw) and may die suddenly. Chronic disease may manifest as decreased appetite, weight loss, and anemia [18]. Young animals usually carry heavier burdens than adult sheep and goats. Occurrence and severity of* H. contortus* infection also depend on the rainfall patterns and temperatures of an area [13]. High rainfall and temperatures promote rapid hatching of eggs on pasture and hence increased contamination.

The prevalence of* H. contortus* infections in a flock provides a good indication of the parasite burdens and the magnitude of production losses. Ambient temperature, environmental humidity, grazing behavior of the host, and quantity and quality of pasture are some factors responsible for the prevalence of gastrointestinal helminths. Outbreaks are most common and severe in warm, humid climates [19]. Other risk factors which include host species, sex, age, body condition, breed [16], and intensity of worm infections affect the development of gastrointestinal parasites [20].

Overall prevalence of* H. contortus* infection in small ruminants has been reported variably by different studies from as low as 52.7 to over 83% [13, 15, 16, 21, 22]. One Ethiopian study reported a prevalence of 67.2% and 56.6% in sheep and goats, respectively [16], indicating greater susceptibility of sheep. For instance, a study in Ethiopia reported prevalence of 90.1-96.5% in sheep and 81.8-100% in goats [13]. In South Africa, the prevalence of* H. contortus* infection in sheep has been reported at 68% using the FAMACHA score and 100% with the Hawkesley microhematocrit reader [23]. The same study reported that* H. contortus* was the most prevalent nematode parasite just as had been reported in an earlier study in that country [1]. Reports from other studies indicate that the prevalence of* H. contortus* infection is always higher in sheep than in goats in the same geographical area. In a study conducted in Rwanda,* Haemonchus* spp. were found to be the predominant parasite species in small ruminants with very high infection rates [3].

There are limited reports on the prevalence of gastrointestinal parasites in Rwanda [3, 21]. The prevalence has been reported to be around 58% in Bugesera district in the Southern Province of Rwanda [21] and 88.9% in kids and 73.3% in lambs in Musanze in the Northern Province of Rwanda [3].

Until today, there are very few reports on the prevalence of nematode parasites in Rwanda and, to the best of our knowledge, no studies have been undertaken to assess the burden* Haemonchus* spp. and its implications on small ruminant production on in Nyagatare. The objectives of this study were to determine the prevalence of* H. contortus *egg shedding and the prevalence of* Haemonchus* spp. infections using coprocultures in sheep and goats from five selected sectors of the Nyagatare district where sheep and goats play an important part in human livelihood and sustenance. Furthermore, the study aimed to determine the significance of parasite infections and recommend strategic preventive and control measures with aim of improving the productivity of this sector.

## 2. Materials and Methods

### 2.1. Study Area

**Figure 1:**
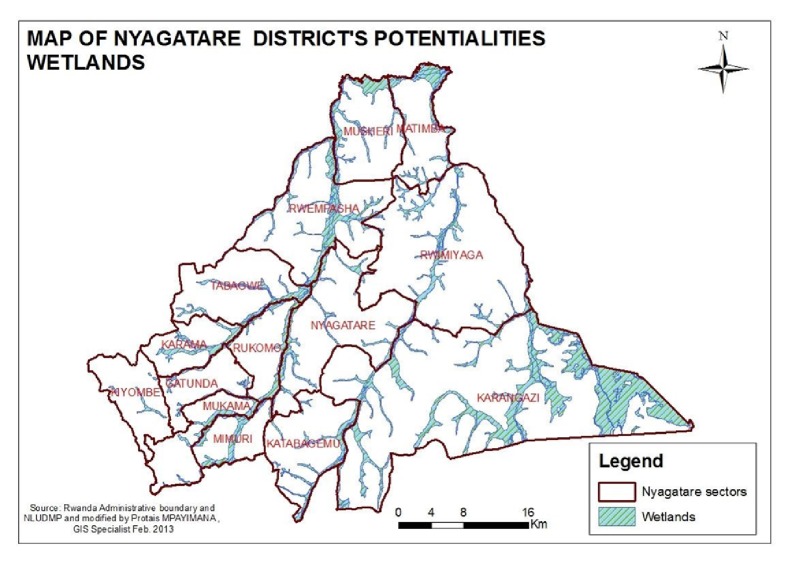
Map of Nyagatare district's potentialities wetlands. Source: http://www.nyagatare.gov.rw/fileadmin/templates/Raports/Nyagatare_Potentialities.pdf.

The study area was in Nyagatare district (1° 18′0′′S, 30° 20′ 0′′E). Of the 14 sectors in Nyagatare, five sectors were chosen for this study: Karangazi in the East, Tabagwe in the West, Matimba in the North, Katabagemu in the South, and Nyagatare in the centre (Figure 1). There are two wet seasons in Nyagatare district; the first is from February to May and the second from September to November. Average precipitation ranges between 1000 mm and 1200 mm annually. Average temperatures vary between 17.8°C and 21°C.

### 2.2. Study Design

A cross-sectional study was used where five sectors were purposively selected out of the 14 sectors of Nyagatare district based on location (i.e., North, South, East, West, and Centre). Selection of sheep and goats from each sector was done biased towards goats whose population is roughly double that of the sheep in Nyagatare district. A random digits table was then used for simple random sampling of animals within each sector. Male and female sheep (local, cross, and South African Meat Merino breeds) and the small East African goat and its crosses with the Alpine, Anglo-Nubian, and Angora goat breeds raised extensively on communal land in the five sectors were sampled [24].

### 2.3. Faecal Sample Collection

Faecal samples were collected per rectum monthly from January to December 2014 from young and adult sheep and goats of all sexes raised on natural pastures in the five sectors. A total of 949 faecal samples were collected from both sheep (n=314) and goats (n=635). Faeces were scooped from the rectum of small ruminants using a gloved index finger in animals restrained in a standing position. Each sample was placed in a separate plastic container with a screw cap, labeled, placed on ice, and transported to the laboratory at the College of Agriculture, Environment, and Veterinary Medicine, University of Rwanda for analysis. Samples that were not tested on the same day were stored at +4°C.

### 2.4. Parasite Identification and Enumeration of* H. contortus* Eggs

Parasite eggs in faecal samples (3 grams) from each animal were isolated using the Modified Wisconsin Sugar Floatation method [25]. Following centrifugation, the supernatant was incubated with Fluorescent-labeled peanut-lectin (*Arachis hypogaea) *agglutinin (Sigma L7381) to identify and enumerate* H. contortus* eggs [26]. Faecal egg counts (FEC) per gram of faeces were performed following the modified McMaster method as described [27] at a sensitivity of 50 eggs per gram (EPG) of faeces.

### 2.5. Statistical Analysis

Descriptive and inferential statistics performed in SPSS version 25. Cross-tabulations were performed to test differences in occurrence of* H. contortus* infections between sectors and within species, age, and sex categories of animals. P values ≤0.05 were considered significant.

## 3. Results

Overall, 75.7% of the small ruminants under study in Nyagatare district (n = 949) had* H. contortus* eggs in faeces. The prevalence of* H. contortus* infection in sheep (83.4%, n = 314) was significantly higher than that in goats of (71.8%, n = 635) (p<0.05).

As shown in Table 1, the overall prevalence of* H. contortus* infection in male and female sheep was 83.5% and 83.4%, respectively, and that in male and female goats was 64.3% and 74.2%, respectively.

As shown in Table 2, significant differences existed in the prevalence of* H. contortus* infection between sheep and goats (Odds Ratio [OR] 1.98, 95% Confidence Interval [CI]: 1.40-2.79, and p≤0.001). There was no difference in the prevalence of* H. contortus* infection when age or sex was considered in sheep. Female goats had a higher prevalence of* H. contortus* infection than male goats (OR 1.60, 95% CI: 1.09-2.36, and p=0.01) and similarly young female goats had a higher prevalence of* H. contortus* infection than young male goats (OR 1.73, 95% CI: 1.01-2.96, and p=0.03).

As shown in Table 3, the prevalence of* H. contortus* infection in sheep from Nyagatare and Karangazi sectors was 88% and 90%, respectively, and that in goats was 85.4% and 89.5%, respectively. The prevalence of* H. contortus* infection in sheep from Tabagwe, Matimba, and Katabagemu sectors was 70.7%, 73.8%, and 72.6%, respectively and that in goats was 55.1%, 64.3%, and 61.5%, respectively.

As shown in Table 4, there was no significant difference in the occurrence of H contortus infections in sheep between the five sectors (p>0.05).

As shown in Table 5, the prevalence of* H. contortus* infection in goats from Nyagatare sector was greater than in goats from Matimba (OR 3.25, 95% CI: 1.76-5.99, and p≤0.001) and Katabagemu sectors (OR 3.67, 95% CI: 2.04-6.59, and p≤0.001). The prevalence of* H. contortus* infection in goats from Karangazi (89.5%) was also greater than in goats from Matimba (OR 4.72, 95% CI: 2.40-9.28, and p≤0.001).

**Figure 2:**
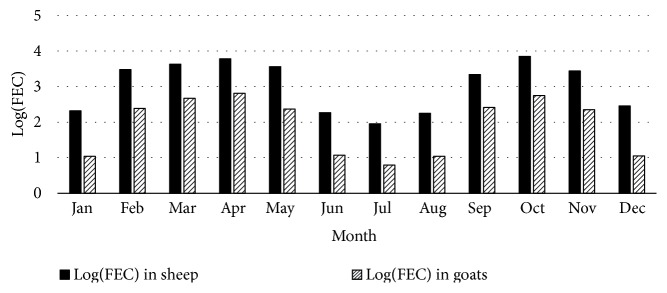
Monthly log (FEC) trends in sheep and goats for the five sectors in Nyagatare district in 2014.

As shown in Figure 2, two distinct periods of higher level of* H. contortus* infection were observed in the first half (February-May) and last half of the year (September-November). A decrease in level of* H. contortus* infection in both sheep and goat was apparent from June to August.

**Figure 3:**
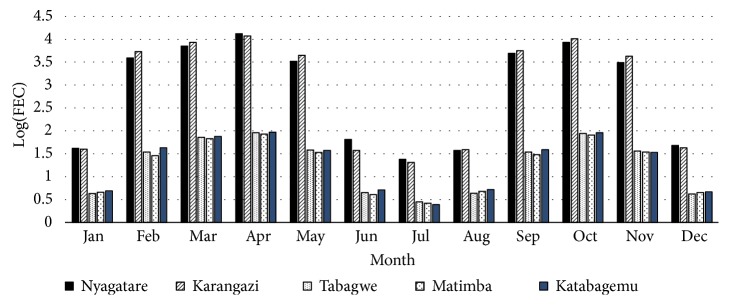
Mean monthly log (FEC) trends for goats per sector in Nyagatare district in 2014.

As shown in Figure 3, a higher level of* H contortus *infection in goats was recorded in all sectors from February to May and from September to November, with a decrease in level of infection from June to August and in December.

**Figure 4:**
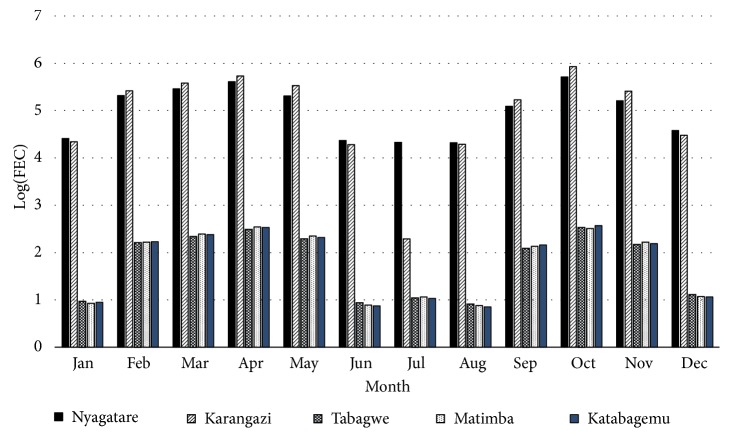
Mean monthly log (FEC) for sheep per sector in Nyagatare district in 2014.

As shown in Figure 4, a higher level of* H contortus *infection in sheep was recorded in all sectors from February to May and from September to November, with a decrease in level of infection from June to August and in December.

## 4. Discussion

Table 1 shows that about 76% of small ruminants in this study shed* Haemonchus* eggs in their faeces. These results indicate a very high level of infection. This high level of infection may partly explain the low productivity of small ruminants in Rwanda as reported in another study [21]. This phenomenon of high nematode prevalence is not new in tropical countries. Similarly high levels of nematode infection like 83.08%, 88.9%, and even 100% have been reported in India, Rwanda, and Pakistan, respectively [3, 22, 28]. In the present study, sheep and goats were raised under an extensive management system, in mostly low-lying pastures where stocking densities were high, nutrition was limited, and veterinary care was almost nonexistent. One study demonstrated a positive correlation between nematode infection and land elevation [28]. These factors promote the exposure of livestock to infective larvae from pastures and the establishment of infections in animals [16].

A significantly higher proportion of sheep (83.4%) than goats (71.8%) had* H. contortus* eggs in their faeces indicating that sheep are more susceptible to* H. contortus* infections than goats. This observation is consistent with the findings of earlier work in Ethiopia [13, 16] that showed higher* H. contortus* infection in sheep than in goats. However, in another study in a different part of Ethiopia, a higher* Haemonchus* spp. infection in goats (71.3%) than sheep (67.57%) was reported [29]. Studies in neighbouring Uganda have reported the prevalence of goats shedding nematode eggs at 53.7% [10] which is lower than that reported in this study. This may be explained by the differences in management practices such as stocking density and ecoclimatic factors. Hot humid areas that receive high rainfall are ideal for reproduction of nematode parasites, contamination of pastures, and survival of infective larvae. Higher stocking densities promote pasture contamination and within-flock transmission of nematode infections. The results of this study contradicted the findings of another study undertaken in Bangladesh which reported a higher occurrence of* H. contortus* infection in goats (35.56%) than in sheep (20%); in that same study, the overall prevalence of gastrointestinal parasitic infection was also found to be higher in goats (45% than in sheep (40%) [30].

The higher prevalence value in sheep than goats arises from the fact that sheep predominantly graze closer to the ground where infective nematode larvae are prevalent, while goats are predominantly browsers and feed on shrubs above the ground where infective larvae are fewer [16]. However, there are a variety of factors which can convolute this expectation. These factors include host age, sex and breeding status, grazing habits, the level of education and economic capacity of farmers, the standard of management, and anthelmintic used [30].

There were no statistically significant differences in the shedding of nematode eggs between male and female sheep (Table 2). These results are in agreement with the findings of a study from Ethiopia [31] and Bangladesh [32]. In contrast, a significantly higher number of female than male goats had nematode eggs in their faeces. Previous studies have shown that female animals are at risk of heavy nematode burdens due to the immunosuppression associated with pregnancy and periparturient periods [7, 15, 16, 22]. In our study, this phenomenon was apparent in goats but not in sheep.

Young animals are generally considered to be more susceptible to nematode infection than adults [6, 32] due to immature active immunity and lack of adaptation in the young [6]. On the other end, immunity also wanes with age. In our study, the differences in nematode infections between adult and young sheep and goats were not statistically significant (Table 2). Older animals would not influence the result much because most families sell the biggest (usually the oldest) animals in order to raise the most money in Rwanda [21]. The finding of having no difference in young than old animals is in agreement with reports from Ethiopia, which indicated that helminth infections affect both young and adult animals equally [31].

The prevalence of sheep and goats that shed nematode eggs in faeces differed by sector of origin. Nyagatare and Karangazi sectors had a significantly higher prevalence of sheep and goats that shed nematode eggs in faeces than sheep and goats from Tabagwe and Matimba and Katabagemu sectors (Figure 4). No significant differences were apparent in the proportions of small ruminants with nematode eggs in faeces between Nyagatare and Karangazi sectors (p<0.05). These findings are explained by the variations local elevation, precipitation, hydrology, and pedology between the sectors. Figure 1shows that Karangazi and Nyagatare both have very extensive hydrological systems and hence flooding and swamp formation is likely. This explains the high infection rates in these 2 sectors. Besides Karangazi is adjacent to a game reserve increasing the likelihood of sharing of parasites with game. On the other hand, Tabagwe, Matimba, and Katabagemu are generally somewhat mountainous and do not promote* H. contortus* infection due to high elevation which favour the growth of browse but not grassland.

Figure 3 shows that faecal egg shedding varied with time of the year in both sheep and goats. Higher faecal egg counts were reported from February to May with a peak in April and from September to November with a peak in October coinciding with the two rainy seasons that are typical of the Nyagatare district. Therefore, rainfall was an important determinant of nematode infection and burden in the study area. Moisture plays a supporting role in the development and survival of the infective stages of nematodes on pasture [33]. Faecal egg counts were significantly higher in the five sectors in February, March, April, and May than in September, October, and November. The lowest FEC were recorded in June, July, August, and December, coinciding with the period of low rainfall.

*Haemonchus contortus eggs* were recovered from 83.4% of sheep and 71.8% goats. Our study focused on* H. contortu*s parasite because it has been reported in many studies as the predominant and most pathogenic of sheep and goat parasite [13, 16, 17] that causes considerable economic losses to the small ruminant industry. Results from coprocultures correlated positively with the faecal egg counts. Our findings are within the range of prevalence of 69.6% and 90.1% reported in sheep and prevalence of 57.1% and 81.8% reported in goats [13]. The overall prevalence of* H. contortus* infection in small ruminants of 75.7% reported in this study is comparable to overall prevalence of 71.03% and 77.38% documented by other studies [13]. This study showed that small ruminants harbored* H. contortus *infections throughout the year with the highest prevalence occurring during the rainy seasons.

The use of chemicals (mainly anthelminthic drugs) has been standard practice for the control of* H. contortus* in ruminants for decades [34]. However the efficacy of this method has been seriously curtailed by the development of anthelminthic drug resistance the world over. This has necessitated the development of alternative techniques such as rotation of anthelminthic class of drugs [35], breeding for anthelminthic resistance [4], vaccination [34], FAMACHA for the implementation of targeted selective treatments (TST), copper oxide wire particles (COWP), nematode trapping fungi, strategic grazing that avoids parasite-laden pastures, phytotherapy [36, 37], and strategic grazing applying the concept of refugia [38].

## 5. Conclusion

Given the high pathogenicity of* H. contortu*s compared with other nematode species, it can be concluded that sheep and goat farmers in the Nyagatare district are at a relatively higher risk of economic loss resulting from reduced productivity, morbidity, and possibly mortality in their flocks. It is well known that* H. contortus* is a problem in hot humid conditions such as occurs in summer in Nyagatare.

## Figures and Tables

**Table 1:** Prevalence of *H. contortus* infectionin small ruminants of Nyagatare district.

**Category **	**Positive animals (**%**)**	**Negative animals (**%**)**	**Total animals (**%**)**
***Sheep***			
Male sheep	111 (83.5)	22 (16.5)	133 (42.4)
Adult male	41 (82.0)	9 (18.0)	50 (15.9)
Young male	70 (84.3)	13 (15.7)	83 (26.4)
Female sheep	151 (83.4)	30 (16.6)	181 (57.6)
Adult female	103 (85.8)	17 (14.2)	120 (38.2)
Young female	48 (78.7)	13 (21.3)	61 (19.4)
**Overall sheep**	**262 (83.4)**	**52 (16.6)**	**314 (100.0)**

***Goats***			
Male goats	99 (64.3)	55 (35.7)	154 (24.3)
Adult male	42 (66.7)	21 (33.3)	63 (9.9)
Young male	57 (62.6)	34 (37.4)	91 (14.3)
Female goats	357 (74.2)	124 (25.8)	481 (75.7)
Adult female	221 (74.2)	77 (25.8)	298 (46.9)
Young female	136 (74.3)	47 (25.7)	183 (28.8)
**Overall goats**	**456 (71.8)**	**179 (28.2)**	**635 (100.0)**

**Total animals **	**718 (75.7)**	**231 (24.3)**	**949 (100.0)**

**Table 2:** Comparison of *H. contortus *infections between and within species.

**Cross-tabulated categories **	**Odds ratio (95**%** C.I.)**	**p-value **
***Between species***		
Sheep versus goats	1.98 (1.40-2.79)	≤0.001*∗*
Adult sheep versus adult goats	2.06 (1.28-3.33)	≤0.001*∗*
Young sheep versus young goats	1.90 (1.16-3.13)	0.01*∗*

***Within sheep***		
Males versus females	1.00 (0.55-1.83)	0.56
Adult females versus adult males	1.33 (0.55-3.22)	0.34
Young males versus young females	1.46 (0.62-3.42)	0.26
Young males versus adult males	1.18 (0.46-3.01)	0.45
Adult females versus young females	1.64 (0.74-3.65)	0.16

***Within goats***		
Females versus males	1.60 (1.09-2.36)	0.01*∗*
Adult females versus adult males	1.44 (0.80-2.57)	0.15
Young females versus young males	1.73 (1.01-2.96)	0.03*∗*
Adult males versus young males	1.19 (0.61-2.34)	0.37
Young females versus adult females	1.01 (0.66-1.54)	0.53

*∗*Significant difference since p≤0.05.

**Table 3:** Prevalence of *H. contortus* infectionin sheep and goats in the five sectors of Nyagatare district.

**Category**	**Nyagatare**	**Karangazi**	**Tabagwe**	**Matimba**	**Katabagemu**	**Total**
***Sheep***						
Animals positive (%)	73 (88.0)	55 (90.2)	41 (70.7)	48 (73.8)	53 (72.6)	262 (83.4)
Animals negative (%)	10 (12.0)	6 (9.8)	11 (19.0)	12 (18.5)	13 (17.8)	52 (16.6)
***Goats***						
Animals positive (%)	117 (85.4)	119 (89.5)	65 (55.1)	72 (64.3)	83 (61.5)	456 (71.8)
Animals negative (%)	20 (14.6)	14 (10.5)	53 (44.9)	40 (35.7)	52 (38.5)	179 (28.2)

**Grand Total **	**194**	**194**	**176**	**177**	**208**	**949**

**Table 4:** Comparison of *H. contortus *infections in sheep between sectors.

**Cross-tabulated sectors **	**Odds ratio (95**%** C.I.)**	**p-value**
Karangazi versus Nyagatare	1.26 (0.43-3.66)	0.45
Nyagatare versus Tabagwe	1.95 (0.77-5.00)	0.12
Nyagatare versus Matimba	1.83 (0.73-4.56)	0.14
Nyagatare versus Katabagemu	1.79 (0.73-4.39)	0.15
Karangazi versus Tabagwe	2.46 (0.84-7.20)	0.08
Karangazi versus Matimba	2.29 (0.80-6.57)	0.09
Karangazi versus Katabagemu	2.25 (0.80-6.35)	0.10
Matimba versus Tabagwe	1.07 (0.43-2.69)	0.53
Katabagemu versus Tabagwe	1.09 (0.44-2.69)	0.51
Katabagemu versus Matimba	1.02 (0.42-2.45)	0.57

**Table 5:** Comparison of *H. contortus* infections in goats between sectors.

**Cross-tabulated sectors **	**Odds ratio (95**%** C.I.)**	**p-value**
Karangazi versus Nyagatare	1.45 (0.70-3.01)	0.20
Nyagatare versus Tabagwe	4.77 (2.63-8.67)	7.61
Nyagatare versus Matimba	3.25 (1.76-5.99)	≤0.001*∗*
Nyagatare versus Katabagemu	3.67 (2.04-6.59)	≤0.001*∗*
Karangazi versus Tabagwe	6.93 (3.58-13.44)	4.72
Karangazi versus Matimba	4.72 (2.40-9.28)	≤0.001*∗*
Karangazi versus Katabagemu	5.33 (2.77-10.23)	5.99
Matimba versus Tabagwe	1.47 (0.86-2.49)	0.10
Katabagemu versus Tabagwe	1.30 (0.79-2.15)	0.18
Matimba versus Katabagemu	1.13 (0.67-1.90)	0.37

*∗*Significant difference since p≤0.05.

## Data Availability

The data used to support the findings of this study are available from the corresponding author upon request.
